# Synthesis and Comparative Evaluation of Novel Cationic Amphiphile C12-Man-Q as an Efficient DNA Delivery Agent In Vitro

**DOI:** 10.3390/molecules23071540

**Published:** 2018-06-26

**Authors:** Gunita Apsite, Irena Timofejeva, Aleksandra Vezane, Brigita Vigante, Martins Rucins, Arkadij Sobolev, Mara Plotniece, Karlis Pajuste, Tatjana Kozlovska, Aiva Plotniece

**Affiliations:** 1 Latvian Biomedical Research and Study Centre, Ratsupites iela 1, LV-1067 Riga, Latvia; gunita.apsite@gmail.com (G.A.); irena@biomed.lu.lv (I.T.); Aleksandra.Vezane@biomed.lu.lv (A.V.); tatyana@biomed.lu.lv (T.K.); 2 Latvian Institute of Organic Synthesis, Aizkraukles iela 21, LV-1006 Riga, Latvia; vigante@osi.lv (B.V.); rucins@osi.lv (M.R.); arkady@osi.lv (A.S.); marapl67@hotmail.com (M.P.); kpajuste@osi.lv (K.P.); 3 Rīga Stradiņš University, Dzirciema iela 16, LV-1007 Riga, Latvia

**Keywords:** cationic amphiphiles, 1,4-dihydropyridines, lipoplexes, DNA transfection, gene delivery, cytotoxicity

## Abstract

New amphiphilic 1,4-DHP derivative **C12-Man-Q** with remoted cationic moieties at positions 2 and 6 was synthesised to study DNA delivery activity. The results were compared with data obtained for cationic 1,4-DHP derivative **D19**, which is known to be the most efficient one among the previously tested 1,4-DHP amphiphiles. We analysed the effects of **C12-Man-Q** concentration, complexation media, and complex/cell contact time on the gene delivery effectiveness and cell viability. Transmission electron microscopy data confirms that lipoplexes formed by the compound **C12-Man-Q** were quite uniform, vesicular-like structures with sizes of about 50 nm, and lipoplexes produced by compound **D19** were of irregular shapes, varied in size in the range of 25–80 nm. Additionally, confocal microscopy results revealed that both amphiphiles effectively delivered green fluorescent protein expression plasmid into BHK-21 cells and produced a fluorescent signal with satisfactory efficiency, although compound **C12-Man-Q** was more cytotoxic to the BHK-21 cells with an increase of concentration. It can be concluded that optimal conditions for **C12-Man-Q** lipoplexes delivery in BHK-21 cells were the serum free media without 0.15 M NaCl, at an N/P ratio of 0.9. Compound **D19** showed higher transfection efficiency to transfect BHK-21 and Cos-7 cell lines, when transfecting active proliferating cells. Although **D19** was not able to transfect all studied cell lines we propose that it could be cell type specific. The compound **C12-Man-Q** showed modest delivery activity in all used cell lines, and higher activity was obtained in the case of H2-35 and B16 cells. The transfection efficiency in cell lines MCF-7, HeLa, and Huh-7 appears to be comparable to the reference compound **D19** and minimal in the HepG2 cell line.

## 1. Introduction

Gene therapy has become the research focus for many laboratories in pharmacy, medicine, biochemistry, and chemical engineering worldwide [1,2]. In gene therapy DNA, RNA, or gene activity controlling elements are used as pharmacological agents to cure diseases by replacing damaged genes with a proper one [3,4,5,6]. Initially, gene therapy was viewed as an approach for treating hereditary diseases, but its potential role in the treatment of acquired diseases, such as cancer, is now widely recognised [7,8,9].

The success of gene therapy is largely dependent on the development of a vector or vehicle that can selectively and efficiently deliver a gene to target cells with minimal toxicity [10,11,12]. The two main types of vectors that are used in gene therapy are based on viral and nonviral gene delivery systems. Though viral systems usually give high transfection efficiency, safety concerns from potential mutation, recombination, oncogenic effect, and high cost, however, greatly limit their therapeutic applications [13,14]. Compared to viral vectors, nonviral gene delivery systems are known to possess a low preexisting immunogenicity. Additional advantages of nonviral vectors are their higher loading capacity and facile preparation methods [15]. Consequently, the development of novel nontoxic cationic structures with high gene transfection efficiencies is of great importance [16].

Synthetic cationic lipids are widely used components of nonviral gene carriers and the factors regulating their transfection efficiency are subject of considerable interest [17]. Synthetic cationic lipids are currently the most extensively used nonviral carriers due to their ability to form complexes with the nucleic acids [18]. The structure of the lipid molecule, for example, the functional nature hydrophobic domain and the charged headgroup, has a significant impact on the particle integrity and morphology and, hence, on transgene efficiency and cytotoxicity [19]. Each lipid has different structural aspects, such as headgroup size and hydrocarbon tail length. These aspects confer distinct characteristics to the lipid/DNA complex, which, in turn, affect association with, and uptake into, the cell [20]. The molecular architecture of the cationic lipid determines its transfection efficiency [21].

Positively-charged amphiphilic 1,4-dihydropyridine (1,4-DHP) derivatives were studied earlier as membranotropic compounds [22] and proposed as a promising tool for DNA delivery [23,24]. Previously, multiple cationic 1,4-dihydropyridine amphiphiles capable of transfecting plasmid DNA (pDNA) into different cell lines in vitro were developed. After these studies we have concluded that two cationic moieties at positions 2 and 6, together with dodecyloxycarbonyl substituents at positions 3 and 5 of the 1,4-DHP molecule, were optimal for these kinds of synthetic lipid-like compounds [23,24]. The obtained data confirmed that, at charge ratio 2, the transfection efficiency of 1,4-DHP amphiphile **D19** was about five times better than that reported for the commercially available cationic lipid of DOTAP (*N*-(1-(2,3-dioleoyloxy)propyl)-*N*,*N*,*N*-trimethyl ammonium methylsulfate). The compound was also 45 times more effective than PEI 25 (polyethyleneimine of 25 kDa) [24]. Recently, the influence of the amphiphile core on the efficacy of pDNA delivery has been revealed within studies of structure-activity relationships performed by our research group. During these studies the activity of the corresponding cationic pyridines as close structural analogues, and possible metabolites of promising 1,4-DHP delivery systems, were examined. The obtained data showed that pyridine derivatives having dodecyloxycarbonyl substituents at positions 3 and 5, and cationic moieties at positions 2 and 6, possessed lower pDNA transfection activity than the corresponding 1,4-DHP amphiphiles [25].

In this study two promising cationic compounds on 1,4-DHP core were studied. Compound **C12-Man-Q** was synthesised for testing of the influence of remoted cationic moieties at positions 2 and 6 of the 1,4-DHP molecule on DNA delivery activity. The design of compound **C12-Man-Q** was based on the exchange of aromatic pyridinium moieties of derivative **D19** to aliphatic ammonium moieties. The results were compared with data obtained for cationic 1,4-DHP derivative **D19**, which is known to be the most efficient one among the 1,4-DHP derivatives tested previously [23,24,25]. In this work we analysed the effects of **C12-Man-Q** concentration, complexation media, and complex/cell contact time on the gene delivery effectiveness and cell viability on various cell lines.

## 2. Result and Discussion

### 2.1. Synthesis of Cationic 1,4-Dihydropyridine Derivatives ***D19*** and ***C12-Man-Q***

**Scheme 1:**
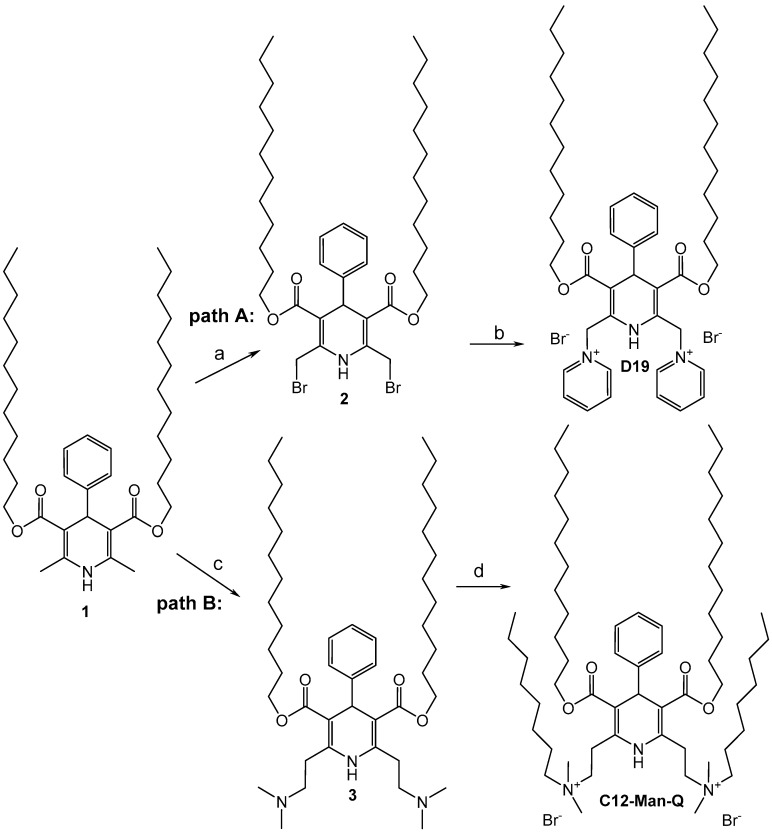
Strategies for the synthesis of cationic 1,4-DHP derivatives **D19** and **C12-Man-Q**. Reaction conditions: (**a**) NBS, MeOH, r.t., 20 h; (**b**) pyridine, dry acetone, r.t. 48 h [26]; (**c**) paraformaldehyde, dimethylamine hydrochloride, EtOH, conc.HCl, reflux, 24 h; and (**d**) 1-bromooctane, nitromethane, DMF, reflux, 48 h.

The reference compound 1,1′-[(3,5-didodecyloxycarbonyl-4-phenyl-1,4-dihydropyridine-2,6-diyl)dimethylen]-bispyridinium dibromide (**D19**) was synthesised according to the already-reported method [23], path A (Scheme 1). The target 1,4-DHP derivative *N*,*N*′-{[3,5-bis(dodecyloxycarbonyl)-4-phenyl-1,4-dihydropyridine-2,6-diyl]diethylene}bis *N*,*N*-dimethyloctyl ammonium dibromide (**C12-Man-Q**) was designed to remain in two cationic moieties at positions 2 and 6 and dodecyloxycarbonyl substituents at positions 3 and 5 of the 1,4-DHP molecule which were defined as optimal for these kinds of synthetic lipid-like compounds as delivery systems according to our previous studies [23,24]. In this case, remoted 1,4-DHP cycle cationic aromatic headgroups were exchanged with aliphatic headgroups at positions 2 and 6 providing compound **C12-Man-Q**. Studies of the differences in activities of **D-19** and **C12-Man-Q** are important steps in establishing the structure-activity relationships and elaboration of more active and selective cationic 1,4-DHP derivatives in the future. The desired cationic 1,4-DHP derivative **C12-Man-Q** was synthesised in the two-stage procedure according to path B (Scheme 1).

2,6-Bis(2-(*N*,*N*-dimethylamino)ethyl) 1,4-dihydropyridine derivative **3** was obtained in 68% yield in the Mannich reaction, analogous with the method reported in the literature [27]. In our case product purification was performed by flash chromatography. It can be assumed that the Mannich reaction occurred with a lower yield due to the presence of didodecyloxycarbonyl substituents at the positions 3 and 5 of 1,4-DHP cycle, compared to the previously-described methoxycarbonyl moieties [27], which were less sterically hindered. Further quaternisation of 1,4-DHP **3** with 1-bromooctane with continuous refluxing in a mixture of nitromethane and DMF led to the target aliphatic moieties containing dicationic 1,4-DHP derivative **C12-Man-Q** in 50% yield.

The structures of the newly-synthesised compounds **3** and **C12-Man-Q** were established and confirmed by ^1^H-NMR, ^13^C-NMR, MS, and elemental analysis data. Details of the syntheses and full physico-chemical characterisation for the newly-synthesised compounds are given in the Experimental section. The purities of the studied compounds were at least 95% according to high-performance liquid chromatography (HPLC) data [28].

### 2.2. Choice of Plasmid DNA

Plasmid DNA (pDNA), as a DNA vector for transferring different genes of interest, has been widely used as gene delivery systems in vitro [29]. Plasmid vectors were classified into different classes in accordance to specificity of existing replication origin, selection markers and promoter sequences [30]. In this study, two plasmid vectors with relatively similar sizes (4079 bp and 4731 bp) and the same, strong constitutive cytomegalovirus (CMV) promoter [31] for transcriptional control of two different reporter genes’ expression were used. Plasmid pRL-CMV (Promega, Madison, WI, USA) was used for delivery of the wild-type Renilla luciferase gene, thus allowing the evaluation of the transfection efficiency quantitatively. Plasmid pEGFP-C1 (Clontech) encoded for a red-shifted variant of wild-type GFP [32,33,34], which has been optimised for brighter fluorescence and higher expression in mammalian cells, was used to assess transfection efficiency visually with fluorescent microscopy. Interpretation of the results is based on the assumption, that although absolute gene expression values vary with the plasmid construct, the relative expression levels for various transfection reagents are related directly to the reagent and not to the plasmid [35,36].

### 2.3. Evaluation of Complexation Ability

Formation of complexes (lipoplexes) between lipids and DNA is defined as the electrostatic interaction between anionic-charged DNA and the cationic-charged gene delivery vehicle. The first step in the gene delivery process is evaluation of the complexation ability of the carrier molecule with the pDNA, followed by pDNA condensation and formation of the carrier/pDNA complex. The N/P ratio refers to ionic balance in cationic amphiphiles/DNA transfection lipoplexes and indicates the ratio between nitrogen (N) from the synthetic lipid and phosphate (P) residues from the DNA. In most cases, in order to achieve higher transfection efficiency, it is crucial for the transfection complex to stay positively charged so that it could interact with cell plasmatic membrane on the same electrostatic forces basis [37,38,39]. Therefore, the relative pDNA binding affinities of the synthesised 1,4-DHP amphiphiles **D19** and **C12-Man-Q** were evaluated using a standard electrophoresis gel shift assay for the assessment of pDNA (pEGFP-C1) complexation at various N/P ratios. Increasing the amounts of the compounds **C12-Man-Q** or **D19** were mixed with a constant amount (1 µg) of plasmid DNA (pEGFP-C1) and analysed by agarose (0.9%, *w*/*v*) gel electrophoresis. The assay is based on the ability of free negatively-charged pDNA to migrate into the gel upon application of an electrical field, whereas complexed pDNA lacks this property. The formation of the **D19**/DNA complexes as evaluated in serum-free cell growth medium while **C12-Man-Q**/DNA complexes were evaluated both in serum free cell growth medium and in the same medium supplemented with NaCl (0.15 M) at various amphiphile/DNA (N/P) ratios. NaCl is an important component of most biological fluids, including serum. Even-Chen et al. demonstrated that for cationic lipid compositions, the addition of NaCl prevented lipoplex formation and induced partial dissociation between lipids and DNA of the preformed lipoplexes [40]. NaCl additive in transfection media was also used in our previous studies for development of 1,4-DHP amphiphiles as pDNA delivery agents and evaluated 1,4-DHP amphiphile **D19** as a perspective delivery system [23,24].

**Figure 1:**
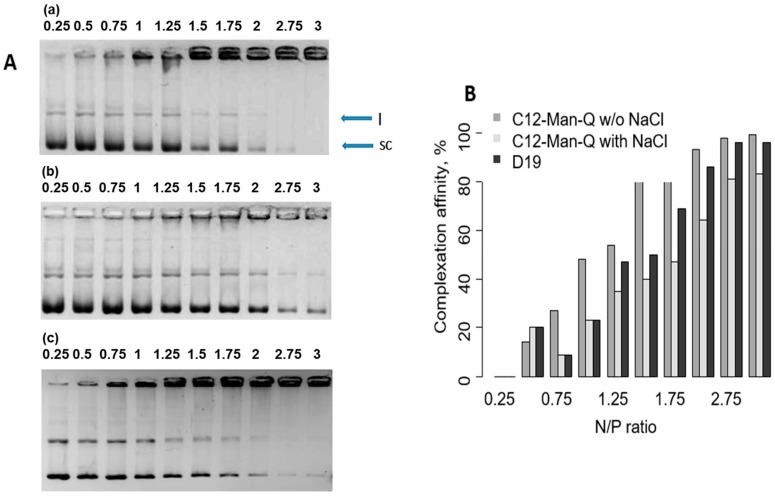
pDNA (pEGFP-C1) and cationic amphiphiles affinity test obtained by gel electrophoresis assay at various N/P (0.25–3) ratios. Supercoiled (sc) and linear (l) forms of plasmid DNA indicated by arrows. (**A**) pDNA retardation assay: (**a**) for **C12-Man-Q**/pDNA complexes without NaCl; (**b**) for **C12-Man-Q**/pDNA complexes with 0.15 M NaCl; (**c**) for **D19**/pDNA complexes with NaCl; and (**B**) quantification of the pDNA binding shift by densitometry (the percentage of the bonded supercoiled DNA was assessed by the density of bands on agarose gel using the open-access image processing program ImageJ) [41].

The binding affinities of the studied 1,4-DHP amphiphiles **D19** and **C12-Man-Q** are represented in Figure 1. The efficiency of pDNA complexation increased, along with growing the amphiphile/DNA complexes’ N/P ratio. The ability to complex the pDNA was approved by evident disappearance of supercoiled and linear forms of the pDNA for both compounds. For compound **D19** almost all pDNA has been bound at N/P ratio of 2, which is in a good agreement with our previous data [23,24]. The effectiveness of 1,4-DHP derivative **C12-Man-Q** in lipoplex formation was influenced by complexation media and decreased up to 20% in the presence of 0.15 M NaCl (Figure 1B). It was confirmed that media significantly affected the complexation ability of compound **C12-Man-Q**, which was in agreement with other group conclusions, that transfection medium properties (ionic strength, counterion species, and pH) were very important and affected cell surface and lipoplex electrostatics [40].

**Figure 2:**
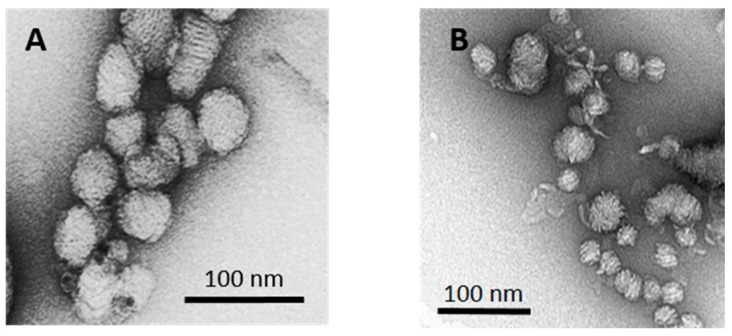
Transmission electron microscopy (TEM) images of lipoplexes formed by cationic lipid and pDNA (pEGFP-C1) at optimised N/P ratio. (**A**) lipoplexes formed by amphiphile **C12-Man-Q** (N/P ratio of 0.9); and (**B**) lipoplexes formed by amphiphile **D19** (N/P ratio of 2). Scale bars represent 100 nm.

Among factors regulating the activity of transfection complexes, the size of the lipoplexes has been reported to be a major determinant of transfection efficiency [42,43,44]. The morphology and size of the lipoplexes formed by 1,4-DHP amphiphiles **C12-Man-Q** and **D19** were analysed by the transmission electron microscopy (TEM) technique. As seen in TEM images (Figure 2), lipoplexes formed by the compound **C12-Man-Q** (Figure 2A) were quite uniform, about 50 nm in size and resembled vesicular-like structures. In turn, lipoplexes produced by compound **D19** (Figure 2B) were of irregular shapes and these complexes varied in their size in the range 25–80 nm.

Obtained data about lipoplex sizes for compound **D19** coincide well with previous results, confirming that compound **D19** and its structural analogues having different alkyl chains at 3,5-positions of 1,4-DHP and pCMV-βGal plasmid containing the β-galactosidase cDNA driven by the CMV promoter formed lipoplexes with diameters in the range below 150 nm [23].

### 2.4. Cytotoxicoty Evaluation of 1,4-DHP/DNA Complexes

**Figure 3:**
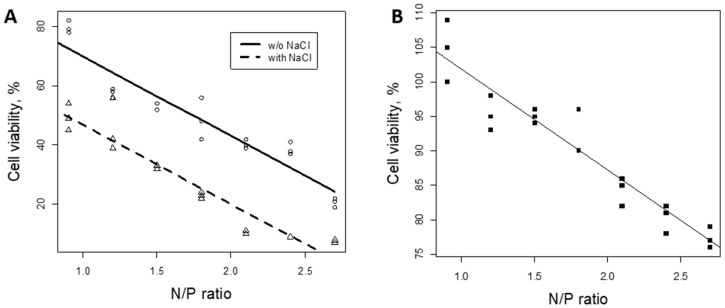
Viability of BHK-21 cells (%) 24 h post transfection determined by MTT assay. (**A**) **C12-Man-Q**/DNA complexes; (**B**) **D19**/DNA complexes. DNA-pEGFP-C1. For each value of the N/P ratio three independent repeats were performed.

The cytotoxic effect of the lipoplexes formed by 1,4-DHP amphiphiles/DNA towards mammalian cells was evaluated using colorimetric 3-(4,5-dimethylthiazol-2-yl)-2,5-diphenyltetrazolium bromide (MTT) assay [45]. The cells were treated with 1,4-DHP amphiphile/DNA complexes at various N/P ratios. Obtained data for the viability of BHK-21 cells are presented in Figure 3.

As shown in Figure 3, linear coherence between the used amount of 1,4-DHP amphiphiles and the viability of BHK-21 cells is evident for both compounds. An increase of the N/P ratio means decreasing cell viability. In general, lipoplexes formed by compound **C12-Man-Q** are more toxic to cells, and the toxicity is increased when NaCl (0.15M) is used as an additive of lipoplex formation medium (Figure 3A). Thus, lipoplexes of compound **C12-Man-Q** showed only 50% cell viability, when the lowest N/P ratio of 1 was used. Cell viability results were improved when the transfection complexes were prepared in medium without the addition of NaCl, giving 50% cell viability at an N/P ratio of 2. In turn, lipoplexes formed by compound **D19** (Figure 3B) showed minimal toxic effect on BHK-21 cells. Moreover, compound **D19**, evidently, does not affect cell division and, as a result, cell viability values 24 h post transfection are even higher (above 100%) than that of control, non-transfected cells.

**Figure 4:**
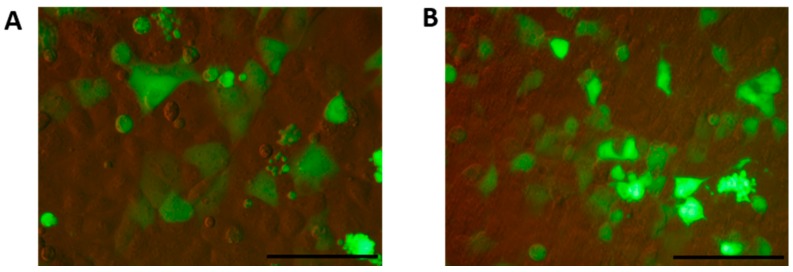
Cos-7 cell morphology evaluated by fluorescent microscopy 24 h post transfection with 1,4-DHP/DNA (pEGFP-C1) lipoplexes. (**A**) **C12-Man-Q**/DNA lipoplexes; and (**B**) **D19**/DNA lipoplexes. Scale bars represent 100 μm.

It should be noted that evaluation of Cos-7 cell morphology after treatment with **C12-Man-Q**/DNA (pEGFP-C1) lipoplexes (Figure 4A) does not reveal any signs of toxicity caused by the used transfection reagent. Therefore, we could assume that different cell types might have different responses to treatment by **C12-Man-Q**/DNA lipoplexes, thus proving that the optimal transfection reagent for each cell line needs to be determined empirically. Synthesis of new series of cationic amphiphiles differing in cationic substituents may lead to the development of new prospective transfection reagents in the future.

### 2.5. Evaluation of Transfection Activity

**Figure 5:**
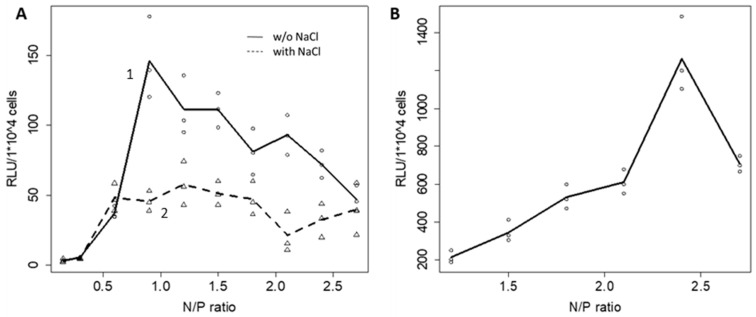
Transfection efficiency achieved using 1,4-DHP amphiphiles determined by luciferase enzyme activity 24 h post transfection. (**A**) Compound **C12-Man-Q** (1-without NaCl; 2-with NaCl additive); and (**B**) compound **D19**. For each value of the N/P ratio three independent repeats were performed.

To evaluate the efficiency of the compounds **C12-Man-Q** and **D19** to deliver plasmid DNA into mammalian cells, first, we performed a transient transfection of BHK-21 cell line. The expression vector pRL-CMV was used to evaluate the transfection efficiency of the new transfection agent **C12-Man-Q** at various N/P ratios. Increasing amounts of **C12-Man-Q** were mixed with 1 μg of pDNA to achieve N/P ratios varying from 0.25 to 3.0, and transfection activity of the lipoplexes was assessed by the luciferin-luciferase reaction test (Renilla luciferase assay system) [46]. Obtained data for transfection activity of 1,4-DHP amphiphiles in BHK-21 cells are presented in Figure 5. According to the obtained data lipoplexes formed by **C12-Man-Q**/DNA in media with NaCl additive demonstrated moderate, yet steady, transfection efficiency beginning from an N/P ratio of 0.5 with no significant drop of the activity along with the increase of N/P ratios up to 3.0 (Figure 5A, curve 2), and in media without NaCl additive, maximal transfection efficiency at an N/P ratio of 0.9, gradually decreasing to an N/P ratio of 3 (Figure 5A, curve 1). By the obtained data **C12-Man-Q** can be characterised as rather inefficient in transfection of BHK-21 cells and, in this case, with completely different action compared with the reference compound **D19**. Obtained data confirmed that the compound **D19** showed dose-dependent incensement of transfection activity at an N/P ratio of 2.4; after that, the activity slowly decreased (Figure 5B). The maximum delivery activity of compound **D19** exceeded the transfection activity of **C12-Man-Q** by almost eight times in BHK-21 cells.

It was also noted earlier that the properties of the transfection medium (ionic strength, counter ion species, and pH) are very important, because of the effect on cell surfaces and lipoplex electrostatics [40,47], which confirms the NaCl additive’s influence on **C12-Man-Q** delivery efficiency.

A promising nonviral transfection gene delivery vehicle should have high transfection activity, but also low cytotoxicity, as cellular damage is an important event accompanying transfection [48,49]. Furthermore, it has long been recognised that different transfection reagents cause different levels of cytotoxicity in different cell lines [50].

**Figure 6:**
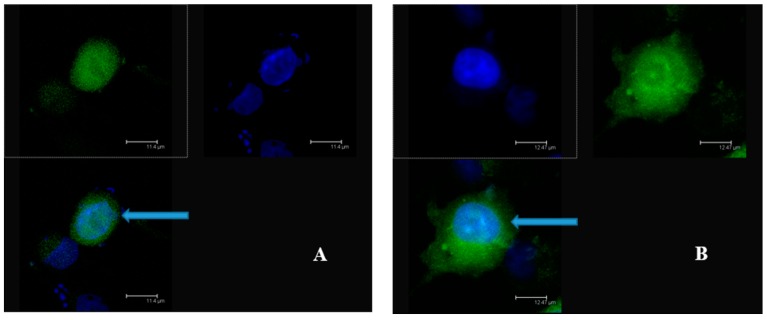
BHK-21 morphology changes (24 h after transfection) under the influence of lipoplexes of **C12-Man-Q** (**A**) and **D19** (**B**) analysed by confocal fluorescent microscopy. Blue (DAPI)—nucleus of the cell, green EGFP expression in transfected cells transfection complex. Scale bars represent 100 μm.

The influence of 1,4-DHP/DNA lipoplexes on BHK-21 cell morphology changes (24 h after transfection) were evaluated by confocal fluorescent microscopy. The obtained data are presented at Figure 6.

Confocal microscopy results revealed that both amphiphiles **C12-Man-Q** and **D19** delivered a green fluorescent protein expression plasmid into BHK-21 cells and produced a fluorescent EGFP signal with satisfactory efficiency. However, for the new compound **C12-Man-Q** cytotoxicity of the preformed transfection complexes (Figure 6A), revealed as distinct morphological changes (cell shrinkage) in transfected BHK-21 cells, was one of the most important factors that negatively influenced 1,4-DHP derivative **C12-Man-Q** transfection potential. Oppositely, the reference compound **D19**, as a part of the transfection complex, did not affect the transfected cell morphology, as evidenced by the predominantly uniform cytoplasmic distribution of EGFP fluorescence in cells (Figure 6B).

**Figure 7:**
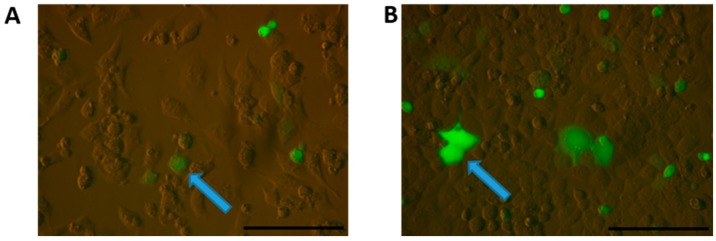
Influence of the ionic strength of the lipoplex formation medium on BHK-21 cell morphology changes 24 h after transfection by **C12-Man-Q**/pEGFP-C1 lipoplexes. (**A**) lipoplexes prepared in medium containing 0.15 M NaCl; and (**B**) lipoplexes prepared in medium without the addition of NaCl. Green cells represent transfected cells, which express pEGFP. Scale bars represent 100 μm.

Simultaneously, we evaluated the influence of ionic strength of the complexation medium and optimised the contact time between the lipoplex and the cell surface on cytotoxicity and transfection efficiency of **C12-Man-Q** lipoplexes. Initially, the influence of ionic strength on BHK-21 cell morphology after transfection by **C12-Man-Q**/pEGFP-C1 lipoplexes were evaluated in the serum free media with and without 0.15 NaCl additive [23]. The obtained data are presented in Figure 7.

Comparison of the morphology of BHK-21 cells (Figure 7) transfected by **C12-Man-Q**/pEGFP-C1 lipoplexes showed the effect of NaCl on cell proliferation. The density of cells in the Figure 7A is about 50% compared to Figure 7B, where the cell density is almost 100%. The NaCl effects on the cells are seen at Figure 7. This effect has resulted in reduced cell viability and also led to the conclusion that increments of ionic strength of complexation medium are the reason for the enhanced toxicity of **C12-Man-Q** for BHK-21 cell (Figure 5 and Figure 6). 

The results revealed that **C12-Man-Q** lipoplexes transfection efficiency on BHK-21 cells could be improved by decreasing the ionic strength of the lipoplex formation medium. As a result transfection efficiency was increased up to three times, when NaCl was removed from the **C12-Man-Q**/DNA complex formation medium (Figure 5A, curve 1). Thus, for an efficient BHK-21 cell transfection the optimal complexation medium for compound **C12-Man-Q** was a serum-free medium without NaCl and the highest transfection activity was achieved when the N/P ratio was 0.9; afterwards, the transfection activity slowly decreased. These conditions were used for the following evaluation of transfection efficiency for compound **C12-Man-Q**.

**Figure 8:**
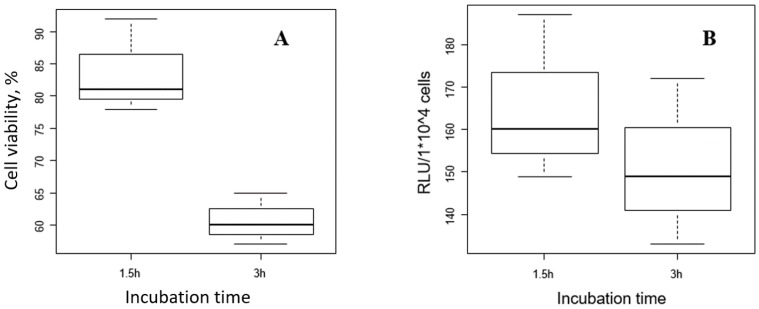
Optimisation of the **C12-Man-Q**/pDNA (pRL-CMV) lipoplexes/cell contact time on BHK-21 cells; N/P ratio of 0.9; sample prepared without additional NaCl. (**A**) The impact of the lipoplexes/cell contact time on the cell viability, determined 24 h post transfection by MTT test; and (**B**) the impact of lipoplexes/cell contact time on transfection efficiency determined by luciferase enzyme activity.

The optimal contact time between lipoplexes and transfecting cells is a rather complex parameter and depends on several factors that influence lipoplex size and stability [51]. Contact time of transfection complexes with cells ranges from 1 h to continuous exposure until the time of analysis [35]. In order to decrease the toxic impact of **C12-Man-Q**-formed lipoplexes on BHK-21 cells, the optimal contact time of lipoplexes and cells was established. We performed correlation analysis to determine whether the reduction of the contact time in half would cause a significant effect on transfection efficiency. The obtained results are presented in Figure 8.

After analysis of the obtained data, it was concluded that there was no significant impact of reducing the incubation time on transfection efficiency (Figure 8B). At the same time the significant improvement of cell viability was observed (Figure 7). Thus, it could be concluded that complexation time for lipoplexes prepared with compound **C12-Man-Q** can be reduced twice without any loss in transfection efficiency, but with a minimisation to lipoplex cytotoxicity.

**Figure 9:**
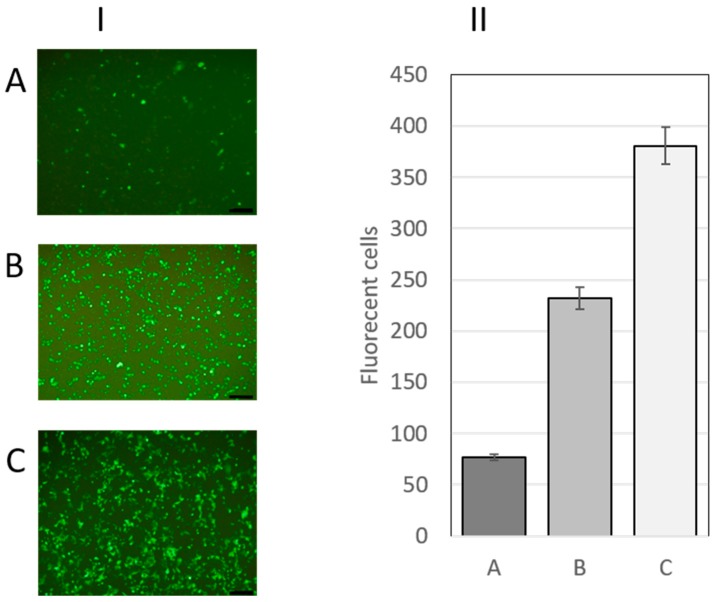
Influence of transfection parameters (lipoplex formation medium and lipoplex/cell contact time) on transfection efficiency of **C12-Man-Q** in BHK-21 cells in comparison to the reference compound **D19**. (**I**): evaluation of EGFP production in cells by fluorescent microscopy (scale bars represent 100 μm): (**A**) transfection by **C12-Man-Q** lipoplexes performed under standard conditions; (**B**) transfection by **C12-Man-Q** after optimisation of the key parameters of the transfection protocol; (**C**) transfection by the reference compound **D19**; (**II**): quantification of the influence of transfection parameters was assessed by amount of fluorescent cells using the open-access image processing program ImageJ.

Optimisation of the key parameters of the transfection protocol allows us to maximize transfection efficiency of the BHK-21 cells by **C12-Man-Q**-formed lipoplexes up to three times (Figure 9).

Optimisation of the key parameters of the transfection protocol demonstrate that the 1,4-DHP derivative **C12-Man-Q** also works as gene delivery agent though still inferior to the reference compound **D19**. Obtained information regarding structure-activity relationships will be helpful for future design of DNA delivery agents on the base 1,4-DHP amphiphiles.

### 2.6. Cell Line-Dependent Differences in Transfection Efficiency of the New Amphiphilic 1,4-DHP Derivative ***C12-Man-Q***

Since transfection efficiency can significantly contrast among different cell lines, it is an absolute perquisite to evaluate new nonviral gene delivery vehicle transfection efficiency in various cell lines. In general, it was underlined that, in many cases, transfection efficiency mediated by nonviral vectors is affected strongly by the cell type [52].

**Figure 10:**
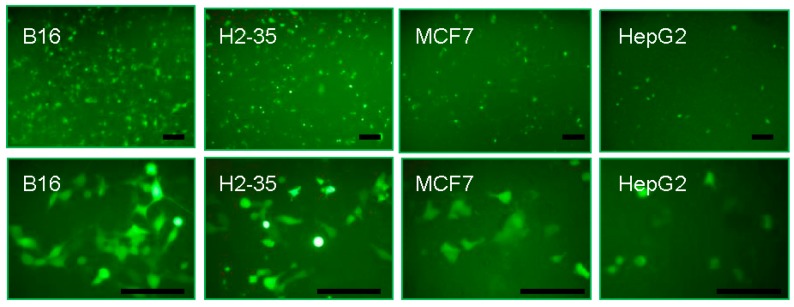
Assessment of transfection efficiency of the **C12-Man-Q** lipoplexes in cancer cell lines by fluorescent microscopy: B16 and H2-35 (both of mouse origin), and MCF-7 and HepG2 (human origin). Scale bars reflect 100 μm.

**Figure 11:**
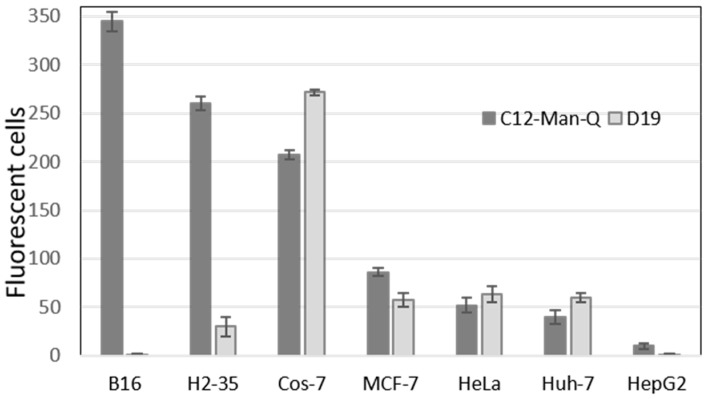
The comparison of the transfection efficiency of the studied amphiphilic 1,4-DHP derivatives **C12-Man-Q** and **D19** in B16, H2-35, Cos-7, MCF-7, HeLa, Huh-7, and HepG2 cell lines. Image analysing program ImageJ was used to count fluorescent cells in the field of view.

Thus, in addition to previously analysed BHK-21 and Cos7 cell lines, for evaluation of transfection ability of the new potential gene delivery agent **C12-Man-Q**, cancer cell lines H2-35, B16 (both of mouse origin), and MCF-7, HeLa, Huh-7, and HepG2 (of human origin) were chosen (Figure 10 and Figure 11). The compound possessed moderate transfection efficiency, but was active in all cell lines used, showing promising potential for in vivo applications. Results were compared with data obtained for derivative **D19** which is the most efficient among the previously studied 1,4-DHP derivatives [23,24,25].

We used the open-access image analysing program ImageJ to count fluorescent cells in the field of view (Figure 11). Compound **D19** showed greater transfection efficiency to transfect cell lines BHK-21 (Figure 9); Cos-7 (Figure 11), but did not succeed in transfecting HepG2 and B16 cell lines. 

The compound **C12-Man-Q** showed rather high delivery activity in the case of H2-35 and B16 cells, while towards other cell lines this compound displayed only modest activity (Figure 11). The transfection efficiency in cell lines MCF-7, HeLa, and Huh-7 was found to be comparable to the reference compound **D19**, while for the HepG2 cell line almost no activity was observed. In general, there should be careful optimisation for every type of cell line because of differences in their metabolic states, lipoplex uptake, and cell trafficking machineries.

## 3. Materials and Methods

### 3.1. General

All reagents were purchased from Acros Organics (Geel, Belgium) or Alfa Aesar (Lancashire, UK) and used without purification. TLC was performed on Silica gel 60 F254 aluminium sheets 20 × 20 cm (Merck KGaA, Darmstadt, Germany). ^1^H-NMR spectra were recorded with a Varian Mercury BB (400 MHz) spectrometer and ^13^C-NMR spectra were recorded with a Varian Mercury BB (100.56 MHz) spectrometer (Agilent, Santa Clara, CA, USA). The coupling constants are expressed in Hertz (Hz). The chemical shifts of the hydrogen and carbon atoms are presented in parts per million (ppm) and referred to the residual signals of the non-deuterated CDCl_3_ (d: 7.25) or partially-deuterated DMSO-*d*_6_ (d: 2.50) solvent for ^1^H-NMR spectra and CDCl_3_ (d: 77.0) or DMSO-*d*_6_ (d: 39.5) solvent for ^13^C-NMR, respectively. Multiplicities are abbreviated as s = singlet; m = multiplet; br = broad. Mass spectral data were determined on a Waters Acquity UPLC system (Waters, Milford, MA, USA) connected to a Waters SQ Detector-2 operating in the ESI positive ion mode on a Waters Acquity UPLC^®^ BEH C18 column (1.7 µm, 2.1 × 50 mm) using a gradient elution with acetonitrile (0.01% trifluoroacetic acid) in water (0.01% trifluoroacetic acid) at a flow rate of 0.5 mL/min. LC-MS data were recorded with a Waters MassLynx 4.1 chromatography data system. Melting points (m.p.) of the synthesised compounds were determined on an OptiMelt (SRS Stanford Research Systems, Sunnyvale, CA, USA). Elemental analyses were determined on an Elemental Combustion System ECS 4010 (Costech Instruments, Pioltello, Italy).

Cell culture BHK-21 (baby hamster kidney fibroblast), Cos-7 (African green monkey kidney fibroblast-like), HeLa (human cervix epithelial cells), HepG2 (human liver epithelial), H2-35 (mouse liver hepatocytes), B16 (mouse melanoma), MCF-7 (human mammary gland adenocarcinoma), and Huh-7 (human hepato cellular carcinoma) cell lines were used in this study. Cells were cultured according to manufacturer’s (American Type Culture Collection (ATCC), Rockville, MD, USA and Japanese Collection of Research Bioresources Cell Bank (JCRB), Osaka, Japan) provided guidelines at 37 °C and 5% CO_2_.

Plasmids**/**pEGFP-C1 (4731 bp) (Clontech, Takara, Tokyo, Japan) was used for most studies (microscopy, complex formation assays). pRL-CMV (4079 bp) (Promega, Fitchburg, WI, USA) was used for transfection efficiency quantification by luciferase reporter assay. Both plasmids were transformed in *Escherichia coli* DH5α and amplified in *E. coli* grown in LB media at 37 °C for 16–18 h. Plasmids were purified by a QIAGEN the EndoFree Plasmid Purification kit (Hilden, Germany). The purified plasmid DNA were dissolved in distilled water and stored at −20 °C. The purity and concentration of plasmid DNA were confirmed by a NanoDrop1000 spectrophotometer (Thermo Fischer Scientific (Waltham, MA, USA), and plasmids DNA were identified by agarose gel electrophoresis.

### 3.2. Synthesis of compounds ***1***, ***2*** and ***D19*** (Scheme 1)

*Compounds***1**, **2***and***D19**. The 3,5-bis(dodecyloxycarbonyl)-2,6-dimethyl-4-phenyl-1,4-dihydro­pyridine (**1**); 2,6-di(bromomethyl)-3,5-bis(dodecyloxycarbonyl)-4-phenyl-1,4-dihydropyridine (**2**) and 1,1′-[(3,5-didodecyloxycarbonyl-4-phenyl-1,4-dihydropyridine-2,6-diyl)dimethylen]-bispyridinium dibromide (**D19**) were synthesised according to the previously-reported methods [23]. Briefly, the elaborated synthesis of the cationic derivative **D19** involves three sequential steps. The first step was a synthesis of the starting 1,4-DHP derivative **1** in a two-component Hantzsch-type cyclisation; the second involved the bromination of the methyl groups 2,6-dimethyl-1,4-DHP derivative **1** with *N*-bromosuccinimide (NBS); and the third step was the nucleophilic substitution of bromine of compound **2** with pyridine yielding the target compound **D19**. ^1^H- and ^13^C-NMR spectra data of compounds **1, 2**, and **D19** were identical to those reported in the literature [26].

*Didodecyl 2*,*6-bis-(2-(N*,*N-dimethylamino)ethyl)-4-phenyl-1*,*4-dihydropyridine-3*,*5-dicarboxylate* (**3**). A mixture of didodecyl 3,5-bis(dodecyloxycarbonyl)-2,6-dimethyl-4-phenyl-1,4-dihydropyridine (**1**, 1.22 g, 2.00 mmol), paraformaldehyde (0.40 g, 0.01 eq, 0.02 mol), dimethylamine hydrochloride (0.98 g, 12.0 mmol), a conc. HCl (0.150 mL) and ethanol (10 mL) was refluxed for 24 h. The solvent was removed by evaporation in vacuo, after which water (5 mL) was added. The pH of the resulting mixture was adjusted to approx. 8 with an aqueous solution of sodium carbonate and extracted with chloroform (6 × 30 mL). The organic layer was washed with brine and dried over MgSO_4_. After removal of the solvent, the oily residue was obtained and purified by flash chromatography (eluent: chloroform:acetone:methanol:Et_3_N = 9:4:2:0.02) yielding 0.75 g (68%) of light yellow foam. TLC: R_f_: 0.2 (CHCl_3_/MeOH/Et_3_N = 1:1:0.01). ^1^H-NMR (CDCl_3_): δ 0.86–0.90 (m, 6H), 1.23–1.34 (m, 36H); 1.55–1.60 (m, 4H), 2.33 (s, 12H), 2.52–2.66 (m, 4H), 2.90–3.09 (m, 4H), 3.98–4.02 (m, 4H), 4.98 (s, 1H), 7.08–7.12 (m, 1H), 7.16–7.20 (m, 2H), 7.26–7.27 (m, 2H), 10.13 (s, 1H) ppm. ^13^C-NMR (CDCl_3_): δ 14.1, 18.4, 22.7, 26.1, 27.8, 28.7, 29.3, 29.4, 29.5, 29.6, 29.7, 31.9, 37.0, 39.4, 44.7, 63.8, 69.2, 102.8, 125.9, 127.7, 127.8, 148.2, 148.3, 167.7 ppm. MS (+ESI) *m*/*z* (relative intensity); 725 ([M + H]^+^, 60). Anal. calcd. for C_45_H_77_N_3_O_4_: C, 74.64; H, 10.72; N, 5.80; Found: C, 74.49; H, 10.55; N, 5.91.

*N*,*N*′*-{[3*,*5-Bis(dodecyloxycarbonyl)-4-phenyl-1*,*4-dihydropyridine-2*,*6-diyl]diethylene}bis N*,*N-dimethyoctyl ammonium dibromide* (**C12-Man-Q**). A mixture of compound **3** (290 mg, 0.4 mmol, 1 eq) and 1-bromooctane (232 mg, 1.2 mmol, 3 eq) in nitromethane (3 mL) and DMF (0.5 mL) was refluxed under argon for 48 h. The solvents were removed in vacuo and the residue was triturated with a small amount of dry acetone. After cooling the precipitate was filtered off and crystallised from dry acetone yielding compound **C12-Man-Q** as a light yellow powder (222 mg, 50%), mp 180–183 °C. ^1^H-NMR (CDCl_3_): δ 0.79–0.84 (m, 12H), 1.21–1.30 (m, 56H), 1.53 (br s, 4H), 1.60–1.87 (m, 8H), 3.22–3.38 (m, 2H)overlap, 3.36 (s, 12H)overlap, 3.48–3.56 (m, 2H), 3.65–3.73 (m, 2H), 3.85–3.97 (m, 4H), 4.24–4.35 (m, 2H), 4.74 (s, 1H), 7.04–7.08 (m, 1H), 7.12–7.15 (m, 2H), 7.20–7.26 (m, 2H)overlap with CDCl_3_, 10.02 (s, 1H) ppm. ^13^C-NMR (CDCl_3_): δ 14.0, 14.1, 22.5, 22.6, 29.3, 29.6, 29.7, 31.9, 39.2, 51.3, 51.8, 61.6, 64.1, 64.7, 105.4, 126.4, 127.9, 128.0, 143.6, 147.2, 166.9 ppm. MS (+ESI) *m*/*z* (relative intensity); 950 ([M-2Br + H]^+^ 10). Anal. calcd. for C_61_H_111_Br_2_N_3_O_4_: C, 65.98; H, 10.08; N, 3.78; Found: C, 65.71; H, 10.15; N, 3.72.

### 3.3. Calculation of Nitrogen to Phosphorus (N/P) Ratio

The N/P ratio is a measure of the ionic balance of the amphiphile/DNA complexes. The positive charge (N) of amphiphile originates from the nitrogens of 1,4-DHP amphiphiles **C12-Man-Q** or **D19**. The negative charge (P) in the plasmid DNA backbone arises from the phosphate group of the deoxyribose nucleotides. The average molecular weight of the nucleotides is assumed to be 330 g/mol.

Example of N/P ratio calculation: Complexation of 1 μg of plasmid DNA with **C12-Man-Q** at N/P ratio 1, how much 0.5 mM of **C12-Man-Q** ($V_{C12\mathrm{Man}Q_{\mathrm{needed}}}$) is needed?

 NP=1×10−6330=3×10−9M=3 nmol

 N=P×1=3×1=3

$$\;V_{C12ManQ_{\mathrm{needed}}}=1\times 3=3\;\mu L$$

### 3.4. Preparation of Amphiphile/pDNA Complexes (Lipoplexes)

The amphiphile/pDNA complexes were prepared using various amounts of cationic lipid and constant amount of pDNA, ensuring different N/P ratios. Firstly, cationic lipid and pDNA were diluted separately in various transfection media: (a) medium without any supplements; and (b) medium without any supplements, with additional 0.15 M NaCl solution; we also compared the complexes’ efficiency made in those media, just with reduced volume. Secondly, after a 5-min long incubation time, diluted pDNA was added by droplets to diluted cationic reagent solution and the whole mixture was then incubated at room temperature for 15 min.

*Agarose gel retardation assay*. The cationic amphiphile/pDNA complexes were prepared by adding 1 μg of DNA to different concentrations of 1,4-DHP derivatives to obtain different N/P charge ratios. After gentle swirling, the mixtures were allowed to stand at room temperature for 10 min prior to analysis. The lipoplexes were prepared in media containing additional 0.15 M NaCl (for compounds **D19** and **C12-Man-Q**) or in media without any additional supplements (for **C12-Man-Q**). After incubation time, gel running buffer with bromophenol blue was added to the complexes and they were loaded on 0.9% agarose gel in TAE buffer, pH 8. Voltage of 65 V was applied for 2.5 h. Following EtBr staining DNA bands were visualised in an UV transilluminator and photographed (BioSpectrum Imaging system, Upland, CA, USA).

### 3.5. In Vitro Transfection Experiment

Cells were seeded onto 24-well plates prior the experiment day with antibiotic-free media so that, at the time of transfection, cells reached 70–80% confluency. The final lipoplexes volume was 200 μL or 100 μL (in case the dilution volume was reduced) and the DNA were used at a concentration of 1 μg/well. Before adding lipoplex solutions, old cell culture medium was removed and cells were washed once with serum-free medium, and the above 200 or 100 μL lipoplexes were added to each well. Plates were incubated for 1.5 to 3 h at 37 °C in a humidified atmosphere containing 5% CO_2_. At the end of the incubation period, into each well fresh medium containing 10% FBS was added. Plates were further incubated for a period of 24 h before evaluating the expression of the reporter gene.

#### 3.5.1. Cytotoxicity Assay

Cytotoxicity of lipoplexes towards used cells was determined using an MTT (3-(4,5-dimethylthiazol-2-yl)-2,5-diphenyltetrazolium bromide) reduction assay. Twenty-four hours post transfection, culture medium was removed and cells were washed once with PBS. Then 270 μL of medium without phenol red and 30 μL of MTT solution (MTT powder was diluted into medium without phenol red in proportion—15 mg powder into 3 mL of medium) was added to each well. The plate was then incubated for 2 h at 37 °C in a humidified atmosphere containing 5% CO_2_. After incubation time 300 μL of MTT solvent (10% Triton X-100, 0.1 N HCl anhydrous isopropanol, 125 mL) was added to each well, and cells were suspended in order to promote dissolution of formed formazan crystals. The absorbance was measured using an FL × 800 fluorescence microplate reader (Biotek, Vernusky, VT, USA) at wavelengths of 570 and 690 nm.

#### 3.5.2. Assessment of Transfection’s Efficiency

For fluorescent microscopy assays, cells were transfected by complexes containing pEGFP-C1. Twenty-four hours after the transfection, the microscope images were obtained at magnifications of 100× and 400×, recorded with Leica DC 150 camera system (Meyer Instruments Inc., Houston, TX, USA) and processed with the open access image processing and analysing program ImageJ.

For luciferase assays, cells were transfected with complexes containing pRL-CMV. For a typical assay in a 24-well plate, 24 h post transfection old medium was removed from the wells and the cells were washed once with PBS. In accordance with the Renilla luciferase assay system (Promega, USA) manufacturer, 100 μL of luciferase lysis buffer was then added to each well, and the cells were lysed for 15 min at room temperature. The cell lysate was transferred to Eppendorf tubes and centrifuged (11,000 rpm for 10 min at 8 °C). For the assay, 20 μL of this supernatant, and 50 μL of luciferase assay substrate (Promega, USA), were used. The luciferase activity was measured in a luminometer Thermo Luminoskan Ascent Reader (Thermo Fischer Scientific, USA) in standard kinetic-luminiscence mode. The integration time of the measurement was 10 s, and a delay of 2 s was given before each measurement. Comparisons of transfection efficiency of various transfection complexes was made based on data for luciferase expressed as relative light units (RLU), normalised for 10,000 cells. All the experiments were done in triplicate, and the results presented are the average of those independent experiments.

#### 3.5.3. Sample Preparation for Confocal Laser Scanning Microscopy

BHK-21 cells were plated onto glass chamber slides for cytochemistry. After 24 h growing cells were transfected with pEGFP-C1 using novel cationic compounds **C12-Man-Q** and **D19**. At 3–24 h post transfection the cells were washed with PBS and fixed with 3.7% formaldehyde (Sigma-Aldrich, USA) for 15 min at +37 °C. Cell nuclei were counterstained with DAPI (c = 4 µg/mL) (Sigma-Aldrich, USA) for 7 min. ProLong^®^ Antifade Kit (P7481) (Molecular Probes/Invitrogen, Eugene, OR, USA) was used for cell mounting. Studies were performed by a Leica DM 6000 B microscope (Leica Microsystems CMS GmbH, Wetzlar, Germany) with a Leica TCS SP2 SE AOBS^®^ laser scanning confocal system (Leica Microsystems CMS GmbH, Wetzlar, Germany).

#### 3.5.4. Sample Preparation for Transmission Electron Microscopy Studies

Samples were prepared as described above (see: Preparation of Liposome/pDNA Complexes (Lipoplexes)), however, DNA and cationic lipids were diluted into the water. The concentration of cationic lipid for electron microscopy was 0.5 μg/μL and, therefore, we calculated the exact amount of pDNA and the volume of cationic lipid. One drop of the sample was adsorbed to a formvar carbon-coated copper grid and negatively stained with 1% aqueous solution of uranyl acetate. The grids were examined with a JEM-1230 TEM (Jeol, Tokio, Japan) at 100 kV.

### 3.6. Statistical Analysis

Data statistical analysis was performed using the open source programming language and software environment for statistical computing and graphics program R [53]. Two sample *t*-test, regression analysis, as well as analysis of covariance (ANCOVA), were performed. Statistical significance was set at *p* < 0.05.

## 4. Conclusions

We tested and compared two novel cationic compounds, **D19** and **C12-Man-Q**, on a 1,4-DHP core as nonviral liposome-based transfection agents. A new amphiphilic 1,4-DHP derivative, **C12-Man-Q**, was synthesised with more remoted cationic moieties at positions 2 and 6 than for the parent compound **D19**. Additionally, instead of aromatic pyridinium moieties, aliphatic ammonium moieties were introduced into the **C12-Man-Q** molecule as cationic headgroups. The results were compared with data obtained for cationic 1,4-DHP derivative **D19**, which is known to be the most efficient one among the previously tested 1,4-DHP amphiphiles. In this study we evaluated the effects of **C12-Man-Q** concentration, complexation media, and complex contact time on the gene delivery effectiveness and cell viability.

Obtained data confirmed that both cationic 1,4-DHP derivatives have the ability to bind pDNA, though their performance of transfecting cells differ significantly. To increase transfection efficiency and reduce toxic cell effects of compound **C12-Man-Q** we found the optimal transfection complex preparation medium (without any supplements) and reduced the transfection complex/cell contact time to 1.5 h. We also explored an optimal N/P ratio of 0.9 for this compound, when transfection efficiency was quite good, and cell viability after transfection was also within the acceptable range.

We evaluated the transfection activity of lipoplexes formed by 1,4-DHP derivatives **D19** and **C12-Man-Q** with pDNA in various cell lines: BHK-21, Cos7, H2-35, B16, MCF-7, HeLa, Huh-7, and HepG2. The most significant property of compound **C12-Man-Q** is its moderate, but still steady, ability to transfect many cell lines, with higher effectiveness in the case of H2-35 and B16 cell lines. The experimental results showed that compound **C12-Man-Q** effectively delivered plasmid DNA into many eukaryotic cell lines, while compound **D19** was defined as cell type specific and possesses higher transfection efficiency in the case of BHK-21 and Cos-7 cell lines.
